# Deep Learning–Based Early Warning Systems in Hospitalized Patients at Risk of Code Blue Events and Length of Stay: Retrospective Real-World Implementation Study

**DOI:** 10.2196/72232

**Published:** 2025-08-22

**Authors:** Ji-hyun Kim, Eun Young Cho, Yuhyun Choi, Joo-Yun Won, Se Hee Cheon, Young Ae Kim, Ki-byung Lee, Kwang Joon Kim, Ho Gwan Kim, Taeyong Sim

**Affiliations:** 1 AITRICS Corp, 218 Teheran-ro, Gangnam-gu, Seoul, 06221, Republic of Korea, 82 025695507, 82 025695508; 2 Department of Medical Management, Presbyterian Medical Center, Jeonju, Republic of Korea; 3 Department of Computing Information, Presbyterian Medical Center, Jeonju, Republic of Korea; 4 Department of Internal Medicine, Chuncheon Sacred Heart Hospital, Chuncheon, Republic of Korea; 5 Department of Internal Medicine, Yonsei University College of Medicine, Seoul, Republic of Korea; 6 Department of Emergency Medicine, Presbyterian Medical Center, Jeonju, Republic of Korea

**Keywords:** adverse event, artificial intelligence, Code Blue, early warning system, rapid response system, VitalCare, hospitalization

## Abstract

**Background:**

In hospitals, Code Blue is an emergency that refers to a patient requiring immediate resuscitation. Over 85% of patients with cardiopulmonary arrest exhibit abnormal vital sign trends prior to the event. Continuous monitoring and accurate interpretation of clinical data through artificial intelligence (AI) models can contribute to preventing critical events.

**Objective:**

This study aims to evaluate changes in clinical outcomes following the use of VitalCare (Major Adverse Event Score and Mortality Score), which is an AI-based early warning system, and to validate the performance of the algorithm.

**Methods:**

A retrospective analysis was conducted by extracting electronic health record data, using a total of 30,785 inpatient cases from general wards and intensive care units. A comparative analysis was performed by setting a 3-month period before and after the system implementation. For clinical evaluation, we measured the incidence rates of Code Blue and adverse events, the proportion of prolonged hospitalization, and the frequency of early interventions. The area under the receiver operating characteristic curve (AUROC) was calculated to assess the performance of the algorithm.

**Results:**

This study demonstrated that, following the implementation of VitalCare, there was a 24.97% reduction in major events such as Code Blue (*P*=.004) and the proportion of prolonged hospitalization in general wards (*P*<.05), along with a significant increase in the rate of early interventions. The model performance exhibited superior outcomes compared with traditional scoring systems, with a Major Adverse Event Score AUROC of 0.865 (95% CI 0.857‐0.873) and Mortality Score AUROC of 0.937 (95% CI 0.931‐0.944).

**Conclusions:**

A well-developed AI-based model that provides high predictive power can contribute to the prevention of major in-hospital events by providing early predictive information to clinicians. Additionally, it plays a crucial role in effectively addressing unmet needs and challenges in terms of human resources and practical procedures.

## Introduction

Code Blue is an emergency code used in hospital settings to indicate a patient experiencing cardiopulmonary arrest (CA), necessitating immediate resuscitative intervention [1]. This represents a critical and resource-intensive process in which health care providers must respond promptly and initiate action as soon as Code Blue is activated. Despite intensive and concerted efforts by medical staff, the survival rate of patients who experience a Code Blue event remains below 20% [1,2]. It is not only directly associated with a poor prognosis and high mortality rate of patients, but also contributes significantly to their economic burden.

Nevertheless, the early identification and prediction of CA or clinical deterioration can be achieved through a comprehensive analysis of patients’ vital signs and blood test results. A previous study demonstrated that over 85% of patients who experience in-hospital CA exhibit abnormal vital signs 6 to 24 hours before adverse events (AEs) [3]. Implementing timely interventions based on these predictions may help delay or prevent mortality. The effective management of Code Blue events depends on the timeliness and quality of interventions [4]. In this context, artificial intelligence (AI)–based early warning systems (EWSs) can facilitate preparedness and support timely intervention by providing advanced alerts. The ability to predict the risk of critical AEs in advance enables hospitals to allocate the necessary resources and establish timely medical treatment plans proactively [1,5].

Numerous studies have demonstrated the positive outcomes of using AI-EWS, underscoring the value of continuous patient monitoring for the early detection of critical events [6-9]. However, most AI-EWS studies have predominantly focused on developing models that predict individual clinical events, such as CA or death, thereby providing limited information. To enhance the early detection of patient deterioration, training algorithms with broadly defined indications are essential. Accordingly, we developed a multilabel algorithm that is designed to recognize the deterioration of patient conditions, including unplanned ward-to-intensive care unit (ICU) transfer (UIT), cardiopulmonary resuscitation, and mortality.

Despite advancements in AI-EWSs, insufficient evidence is available regarding their clinical utility when they are implemented in real-world hospital settings, particularly in terms of reducing in-hospital Code Blue events. Comprehensive validation across multiple domains is essential for the successful implementation and integration of AI-EWS technologies in clinical practice. Therefore, the primary objective of this study was to evaluate the effectiveness of applying an AI-EWS on clinical outcomes, including Code Blue events in hospitals, prolonged length of stay (pLOS), and the frequency of ordering new codes, such as early interventions or assessments for AEs, compared with a silent period using real-world electronic medical record (EMR) data. The secondary objective was to assess the performance in a hospital environment with different characteristics from those of the development data to verify the generalization of the model.

## Methods

### Study Design and Participants

This retrospective study was conducted at the Presbyterian Medical Center in the Republic of Korea, where a rapid response system (RRS) had not yet been established. Instead, the condition of each patient was monitored by an assigned registered nurse for potential clinical deterioration. In March 2023, the hospital introduced an AI solution named VitalCare (VC) for continuous patient monitoring in the general ward (GW) and ICU to predict patient deterioration. The study population consisted of patients aged 19 years and older who were admitted to the GW or ICU over a 17-month period from December 2022 to May 2024. A “silent period” was designated from December 2022 to February 2023 to establish control settings, during which health care providers did not use VC. Excluding the first 1-month adaptation period following the introduction of the system, data from April 2023 to May 2024 were set as the “entire alert period.” We classified and analyzed the intervention period into two categories. To control for seasonal bias, the alert period was defined as the winter months between December 2023 and February 2024 to match the control period, which was also confined to the winter months. Winter was associated with a higher incidence of AEs and mortality. Therefore, this study was designed to focus on comparative analyses specific to winter. To evaluate the overall trend in Code Blue occurrences, a comparative analysis was conducted using 5 years of event data. This analysis compared the 4-year average before the implementation of VC with data from the first year after implementation.

### Ethical Considerations

The Institutional Review Board of the Presbyterian Medical Center approved this study on December 12, 2023, and waived the requirement for informed consent given the specific nature of the retrospective study (2023-12-051). This study was conducted in accordance with the principles of the 1975 Declaration of Helsinki. To protect the privacy of participants, all data were anonymized during extraction from the hospital’s EMR system and used solely for analysis purposes. As this was a retrospective study, no compensation was provided to participants.

### Algorithm Description

The VC algorithm is a deep-learning-based model that uses a bidirectional long short-term memory architecture as a binary classifier to predict the occurrence of major AEs (positive or negative) in GW inpatients. It comprises VC-Major Adverse Event Score (MAES) and VC-Mortality Score (MORS), both of which have been approved by the Korean Ministry of Food and Drug Safety. The VC-MAES is designed to comprehensively predict 3 key events, namely, UIT, death, or cardiopulmonary resuscitation, within the GW. In contrast, the VC-MORS is designed to provide a score that could predict death in patients who are admitted to the ICU. ICU transfers within 3 hours of surgery completion or 12 hours of procedure initiation were considered as planned ICU transfers and were excluded from the outcome analyses. The VC-MAES was trained using data from 334,185 hospitalizations of 209,825 adult patients between 2013 and 2017 at Yonsei Severance Hospital, which is a 2454-bed tertiary academic medical center in Seoul, Republic of Korea. The VC-MORS was trained using data from 21,186 hospitalizations of 19,570 adult patients who were admitted to the ICU between 2013 and 2017 at the same hospital. The dataset encompasses more than 35 surgical and medical specialties. The details of the model architecture were the same as those described in a previous study, with slight modifications [10].

The model primarily uses 5 vital signs—systolic and diastolic blood pressure, heart rate, respiratory rate, and body temperature—along with age to generate risk scores ranging from 0 to 100. Higher scores indicate an increased risk of target events occurring within the next 6 hours. When available, the model also incorporates optional variables, including oxygen saturation (SpO_2_), mental status (Glasgow Coma Scale), total bilirubin, lactate, creatinine, platelet count, pH, sodium, potassium, hematocrit, white blood cell count, bicarbonate, and C-reactive protein. These inputs are updated whenever new data are recorded in the EMR system to ensure that the score reflects the patient’s most recent condition. Given that these optional parameters are infrequently measured, missing values are imputed using the last observation carried forward method, where the most recent available value is used. If no prior values are available, normal reference values are assigned for score computation [11].

### Practical Setting

**Figure 1.**
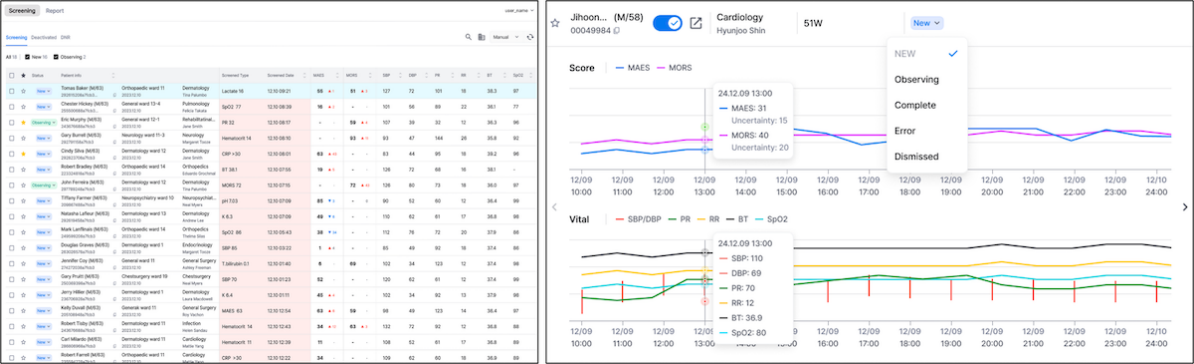
Web interface of VitalCare.

We integrated the hospital network’s EMR system with the pretrained VC algorithm, which was deployed to another institution without any retraining or recalibration, thereby enabling validation using real-world data from hospitalized patients. The system was configured to allow VC access across all GWs and ICUs. Health care providers reviewed single parameters, such as vital signs, laboratory data, and VC prediction scores, for all hospitalized patients via the VC web platform. The VC web platform presents a trend graph of score changes over time, alarm history, and single-parameter history (Figure 1). It was displayed continuously, allowing the health care providers to access it at any time, and clinicians used the information provided by the VC as a reference during clinical decision-making.

### Clinical Evaluation

We analyzed clinical variables such as the in-hospital Code Blue incidence rate, AE (death and UIT cases) incidence rate, proportion of long-term hospitalization during each period, and frequency of early interventions to evaluate the clinical effectiveness following the implementation of VC.

We investigated the annual number of Code Blue events from 2019 to 2023 and calculated the incidence rate relative to the total number of hospitalized patients, excluding those with documented “Do Not Resuscitate” status. The silent period was represented by the average values from 2019 to 2022. The pLOS was defined as the 75th percentile of the total length of stay in the study population [12,13]. Based on this criterion, patients who were admitted to the GW for 9 days or more and those who were admitted to the ICU for 4 days or more were classified as having a prolonged hospital stay. The proportion of patients meeting these criteria was subsequently calculated to evaluate the prevalence of prolonged hospital stay within the cohort.

Given the varying total number of hospital admissions across different periods, the incidence rate of AEs was calculated per 1000 admissions. The number of interventions or evaluations performed within 2 hours of the first alarm activation was calculated to analyze the frequency of early interventions. For this analysis, intervention and evaluation items were compared using prescription codes, such as new oxygenation or change orders, laboratory tests, and any new code orders.

### Performance Evaluation

The overall accuracy of the scores was determined by calculating the area under the receiver operating characteristic curve (AUROC) and area under the precision-recall curve (AUPRC) based on whether a target event occurred within 6 hours of the predicted scores. The bootstrap method was used to compare the AUROC and AUPRC with the National Early Warning Score (NEWS), Modified Early Warning Score (MEWS), Sequential Organ Failure Assessment (SOFA), and Acute Physiology and Chronic Health Evaluation II (APACHE II). Given the extensive use of NEWS, MEWS, and SOFA in studies worldwide to predict cardiac arrest or clinical deterioration, these scores were also used as comparators in this study [1,2,14,15]. NEWS and MEWS were calculated using current values of systolic blood pressure, heart rate, respiratory rate, body temperature, mental status, SpO_2_, and the fraction of inspired oxygen (FiO_2_) recorded in the EMR within 1 hour before the prediction point. The SOFA score was calculated using the worst recorded values in the EMR within 24 hours following ICU admission. As the APACHE II score is specifically used to predict the mortality risk of acutely ill patients within 24 hours of ICU admission [16], the AUROC and AUPRC were calculated based on whether death occurred during the ICU stay.

### Statistical Analysis

Demographic characteristics during the silent and alert periods were compared using chi-square tests for categorical variables. For continuous variables, normality was assessed using the Shapiro-Wilk test and histogram inspection, which noted that large sample sizes may cause the test to reject normality due to high sensitivity. Normally distributed variables were compared using independent 2-tailed *t* tests, and nonnormally distributed variables were compared using the Mann-Whitney *U* test (2-tailed).

To evaluate predictive performance, 95% CIs for the AUROC were estimated using the bootstrap method with 1000 resamples. DeLong’s test was used for statistical comparison of AUROC values, while a bootstrap-based test with 1000 resamples was applied for AUPRC.

Sensitivity, specificity, positive predictive value, and negative predictive value were calculated to evaluate the performance of the scores, using predefined thresholds corresponding to moderate and high alarm levels. Proportions were compared using the 2-sample proportion test, applying either 1- or 2-tailed approaches as appropriate. One-tailed tests were used to compare AE rates when a directional effect was prespecified, whereas 2-tailed tests were applied when no prior direction was assumed.

All statistical analyses were performed using Python (version 3.12.9; Python Software Foundation), with statistical significance defined as *P*<.05, applied uniformly across 1- and 2-tailed tests. The analyses used the MLstatkit and scikit-learn libraries.

## Results

### Overview

**Figure 2.**
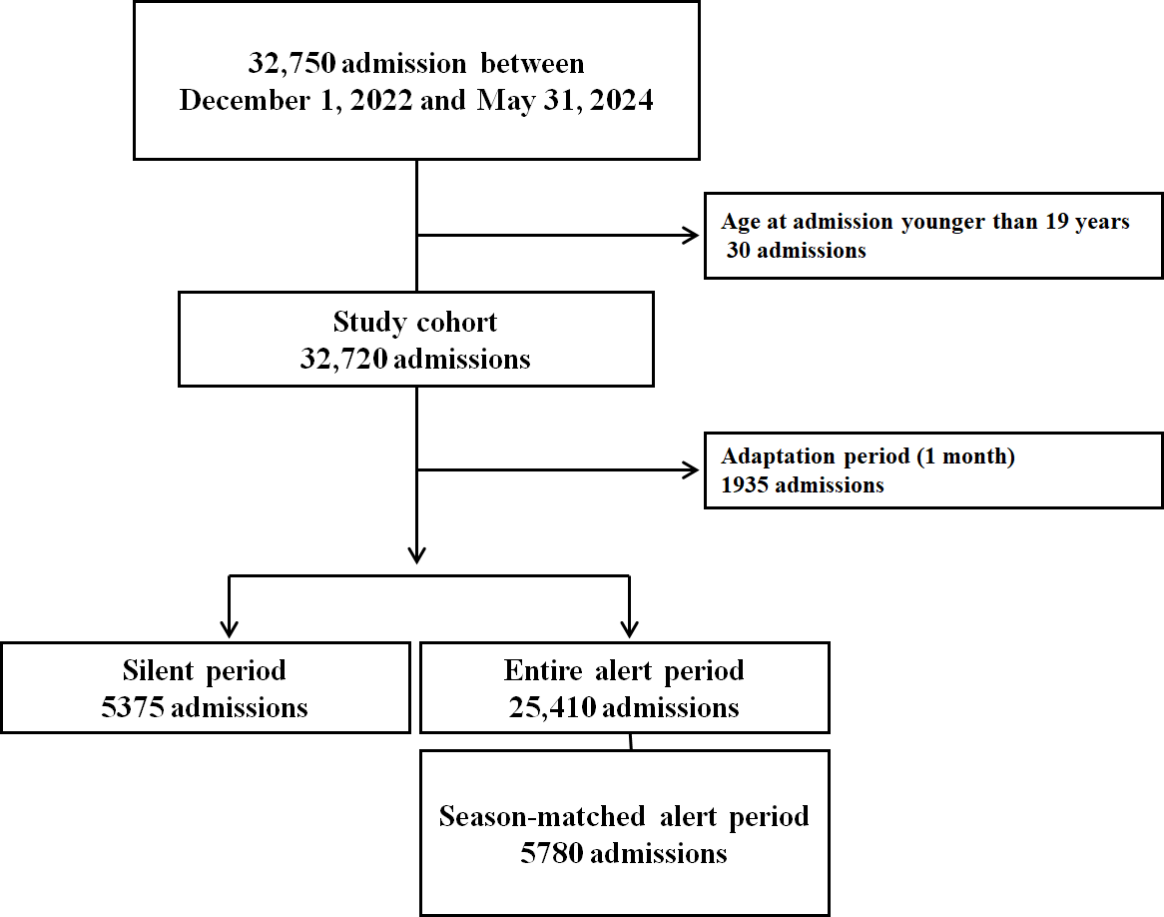
Flowchart of admission selection.

This study initially included 32,750 admissions from 24,187 patients. After applying the exclusion criteria, 30 admissions involving patients aged younger than 19 years of age were excluded, resulting in 32,720 admissions being included in the study dataset, which was used for performance evaluation. Considering the adaptation period, 1935 admissions were excluded from the study. The final dataset consisted of 5375 admissions during the silent period, 25,410 admissions during the alert period, and 5780 admissions during the seasonally matched alert period. There were 3576 ICU admissions, of which 2 were younger than 19 years of age and were excluded, leaving 3574 admissions for the VC-MORS performance evaluation. After excluding 214 admissions during the adaptation period, 3360 admissions remained, including 647 admissions during the silent period, 2713 admissions during the entire alert period, and 597 admissions during the seasonally matched alert period (Figure 2).

### Baseline Characteristics

We compared baseline characteristics of patients admitted during the silent and alert periods, including vital signs, laboratory values, and the Charlson comorbidity index, which was calculated according to previously published methods [17,18]. While statistically significant differences were observed in pulse rate, respiratory rate, SpO_2_, bilirubin, sodium, NEWS, MEWS, Charlson Comorbidity Index, and other baseline characteristics were similar (Table 1). These findings indicate that patient baseline characteristics were largely similar between the 2 periods.

**Table 1.** Characteristics of patients across hospitalizations.

Variables		Overall (n=11,155)	Silent period (n=5375)	Season-matched alert period (n=5780)	Missing, n (%)	*P* value
Age (years), median (IQR)		67.0 (55.0-77.0)	67.0 (55.0-77.0)	67.0 (55.0-77.0)	0 (0)	.90
Sex, n (%)						.19
Female		5632 (50.5)	2679 (49.8)	2953 (51.1)	0 (0)	
Male		5523 (49.5)	2696 (50.2)	2827 (48.9)	0 (0)	
BMI (kg/m^2^), mean (SD)		24.3 (4.1)	24.3 (4.0)	24.3 (4.2)	1787 (16.02)	.58
Vital signs						
Diastolic blood pressure (mm Hg), mean (SD)		76.7 (12.1)	76.9 (12.0)	76.6 (12.2)	4 (0.04)	.27
Pulse (beats per min), mean (SD)		79.4 (14.7)	79.1 (14.7)	79.8 (14.7)	4 (0.04)	.01
Respiratory rate (breaths per min), median (IQR)		20.0 (18.0-20.0)	20.0 (19.0-20.0)	20.0 (18.0-20.0)	4 (0.04)	<.001
Systolic blood pressure (mm Hg), mean (SD)		127.4 (20.1)	127.7 (20.4)	127.1 (19.9)	4 (0.04)	.13
SpO_2_ (%), median (IQR)		97.0 (96.0-98.0)	97.0 (96.0-98.0)	97.0 (96.0-98.0)	2634 (23.61)	.03
Temperature (°C), mean (SD)		36.8 (0.4)	36.8 (0.4)	36.8 (0.4)	6 (0.05)	.18
Laboratory values						
Bilirubin (mg/dL), median (IQR)		0.5 (0.4-0.8)	0.5 (0.4-0.8)	0.5 (0.4-0.8)	2928 (26.25)	<.001
Creatinine (mg/dL), median (IQR)		0.8 (0.6-1.0)	0.8 (0.6-1.0)	0.8 (0.6-1.0)	2362 (21.17)	.15
C-reactive protein (mg/dL), median (IQR)		0.7 (0.1-3.9)	0.6 (0.1-3.7)	0.7 (0.1-4.2)	3605 (32.32)	.24
HCO_3_ (mmol/L), mean (SD)		24.4 (5.7)	24.6 (5.5)	24.3 (5.8)	9437 (84.60)	.21
Hematocrit (%), mean (SD)		35.7 (6.0)	35.6 (6.1)	35.7 (6.0)	1719 (15.41)	.21
Lactate (mmol/L), median (IQR)		2.0 (1.2-4.3)	2.2 (1.3-4.0)	1.9 (1.1-4.3)	10,890 (97.62)	.51
PCO_2_ (mm Hg), mean (SD)		38.3 (10.2)	38.4 (10.0)	38.2 (10.3)	9437 (84.60)	.69
pH, mean (SD)		7.4 (0.1)	7.4 (0.1)	7.4 (0.1)	9437 (84.60)	.17
Platelets (10^3^/µL), mean (SD)		218.9 (80.6)	218.7 (81.5)	219.0 (79.7)	1719 (15.41)	.87
PO_2_ (mm Hg), median (IQR)		92.0 (78.0-113.0)	91.0 (78.0-114.0)	93.0 (78.0-112.0)	9437 (84.60)	.85
Potassium (mEq/L), mean (SD)		4.1 (0.6)	4.0 (0.6)	4.1 (0.6)	2732 (24.49)	.01
Sodium (mEq/L), median (IQR)		141.0 (138.0-142.0)	141.0 (139.0-142.0)	140.0 (138.0-142.0)	2728 (24.46)	.001
White blood cell count (10^3^/µL), median (IQR)		7.0 (5.5-9.2)	6.9 (5.5-9.2)	7.0 (5.5-9.3)	1719 (15.41)	.41
Baseline scores, median (IQR)						
NEWS^a^		3.0 (3.0-4.0)	3.0 (3.0-4.0)	4.0 (3.0-5.0)	549 (4.92)	<.001
MEWS^b^		1.0 (1.0-1.0)	1.0 (1.0-1.0)	1.0 (1.0-1.0)	549 (4.92)	.05
APACHE II^c^		13.0 (9.0-18.0)	13.0 (9.0-18.0)	13.0 (10.0-18.0)	9911 (88.85)	.23
CCI^d^ (comorbidity)		3.0 (2.0-5.0)	3.0 (2.0-5.0)	3.0 (2.0-5.0)	0 (0)	.58

a NEWS: National Early Warning Score

b MEWS: Modified Early Warning Score

c APACHE II: Acute Physiology and Chronic Health Evaluation II

d CCI: Charlson Comorbidity Index

### Reduction in Code Blue and AEs

The incidence of Code Blue was expected to decrease in 2023 compared to the 4-year average between 2019 and 2022. Since the implementation of the VC system in 2023, the hospital’s Code Blue incidence rate (per 1000) has decreased by 24.97%, from 10.57 to 7.93 (*P*=.004). As shown in Table 2, even year-by-year comparisons during the pre-VC period show that the incidence was lower after the introduction of VC.

**Table 2.** Code blue trends before and after VC^a^ implementation.

Year	Number of hospitalized patients^b^	Number of Code Blue events	Code Blue period incidence rate (per 1000)
Before VC implementation			
2019	22,262	262	11.69
2020	21,726	229	10.34
2021	22,087	210	9.32
2022	21,381	223	10.19
Average	21,864	231	10.57
After VC implementation			
2023	21,061	167	7.93

a VC: VitalCare

b Excluding DNR (Do Not Resuscitate) patients.

Moreover, we compared the rates of UIT and in-hospital mortality across three intervals: the silent (pre-VC) period, the season-matched period, and the entire study period. The baseline UIT event rate during the silent period was 69.40 events per 1000 admissions, which significantly decreased to 60.73 (*P*=.03) in the season-matched period and 62.37 (*P*=.03) in the entire study period. For in-hospital mortality, the baseline rate was 19.72 deaths per 1000 admissions, decreasing to 16.78 (*P*=.12) during the season-matched period and further to 15.87 (*P*=.02) during the entire period (Table 3).

**Table 3.** Number of adverse events by period.

	UIT^a^	*P* value	Mortality	*P* value
Silent period	69.40	—^b^	19.72	—
Season-matched alert period	60.73	.03	16.78	.12
Entire alert period	62.37	.03	15.87	.02

a UIT: unplanned ward-to-intensive care unit transfer.

b Not applicable.

### Clinical Outcomes

The pLOS rate in the GW decreased significantly following VC implementation. No significant difference in the pLOS rate was observed before and after the implementation of the VC system in the ICU (Table 4).

**Table 4.** Comparison of length of stay between periods.

	General wards (n=30,785 admissions)		Intensive care units (n=4013 transfers)	
	Proportion of pLOS^a^ (LOS ≥9 days), %	*P* value	Proportion of pLOS (LOS ≥4 days), %	*P* value
Silent period	26.85	—^b^	28.91	—
Season-matched alert period	24.65	.01	30.96	.39
Entire alert period	25.46	.03	28.54	.84

a pLOS: prolonged length of stay

b Not applicable.

As shown in Tables5  6, significantly more early interventions such as oxygenation, laboratory tests, and new code orders occurred following the activation of both moderate (VC-MAES=30) and high (VC-MAES=50) alarms. However, in the ICU, significant earlier interventions were observed only following a moderate VC-MORS alarm (VC-MORS=20), particularly with the ordering of laboratory tests, such as that for lactate.

**Table 5.** Early interventions measure for the first VC^a^ alarm in general wards.

Intervention	Silent period	Season-matched alert period	*P* value	Entire alert period	*P* value
Moderate alarm^b^ (%), n	557	541		2291	
New oxygenation ordered	3.94	9.43	<.001	9.60	<.001
Laboratory tests ordered	10.05	13.86	.05	13.84	.01
New code ordered	24.42	30.87	.02	31.25	<.001
High alarm^c^ (%), n	212	215		870	
New oxygenation ordered	3.77	17.67	<.001	13.10	<.001
Laboratory tests ordered	12.26	21.86	.01	19.43	.01
New code ordered	35.85	47.44	.02	42.76	.07

a VC: VitalCare.

b VC-MAES=30.

c VC-MAES=50.

**Table 6.** Early interventions measure for the first VC^a^ alarm in intensive care units.

Intervention	Silent period	Season-matched alert period	*P* value	Entire alert period	*P* value
Moderate alarm^b^ (%), n	152	141		638	
New oxygenation ordered	12.50	17.02	.28	16.14	.29
Laboratory tests ordered	24.34	35.46	.04	37.15	.002
Lactate ordered	2.63	9.93	.01	7.68	.01
New code ordered	55.26	63.83	.14	62.85	.09
High alarm^c^ (%), n	51	47		214	
New oxygenation ordered	5.88	14.89	.17	9.35	.60
Laboratory tests ordered	27.45	36.17	.36	28.04	.99
Lactate ordered	1.96	6.38	.35	6.54	.31
New code ordered	72.55	72.34	.98	67.29	.51

a VC: VitalCare.

b VC-MORS=20.

c VC-MORS=80.

### Model Performance Compared With Traditional Scoring System

VC-MAES and VC-MORS outperformed conventional EWS in both AUROC and AUPRC. VC-MAES achieved an AUROC of 0.865 (95% CI 0.857-0.873) and an AUPRC of 0.088 (95% CI 0.080‐0.098), which were significantly higher than those of NEWS (AUROC=0.804; AUPRC = 0.018) and MEWS (AUROC=0.772; AUPRC=0.020).

Similarly, VC-MORS showed strong predictive performance for 6-hour mortality, with an AUROC of 0.937 (95% CI 0.931‐0.944) and an AUPRC of 0.098 (95% CI 0.087-0.109), which outperformed SOFA (AUROC=0.754; AUPRC=0.011). APACHE II, calculated once within 24 hours of ICU admission, showed an AUROC of 0.814 and an AUPRC of 0.259 for predicting ICU mortality (Table 7).

**Table 7.** Performance comparison by AUROC^a^ and AUPRC^b^.

Score	AUROC (bootstrap 95% CI)	AUPRC (bootstrap 95% CI)
General wards		
VC^c^-MAES^d^	0.865 (0.857‐0.873)	0.088 (0.080‐0.098)
NEWS^e^	0.804 (0.797‐0.811)^f^	0.018 (0.015‐0.021)^g^
MEWS^h^	0.772 (0.764-0.780)^f^	0.020 (0.015‐0.025)^g^
Intensive care units		
VC-MORS^i^	0.937 (0.931‐0.944)	0.098 (0.087‐0.109)
SOFA^j^	0.754 (0.742-0.765)^f^	0.011 (0.010‐0.012)^g^
APACHE II^k^	0.814 (0.787‐0.840)	0.259 (0.215‐0.307)

a AUROC: area under the receiver operating characteristic curve.

b AUPRC: area under the precision-recall curve.

c VC: VitalCare.

d MAES: Major Adverse Event Score.

e NEWS: National Early Warning Score.

f *P*<.001; Delong test.

g *P*<.001; bootstrap-based test.

h MEWS: Modified Early Warning Score.

i MORS: Mortality Score.

j SOFA: Sequential Organ Failure Assessment.

k APACHE II: Acute Physiology and Chronic Health Evaluation II.

## Discussion

### Principal Findings

We retrospectively collected the EMR data of hospitalized patients and compared the pre- and postintervention clinical outcomes to evaluate the effectiveness of VC. The results demonstrated that VC use was associated with high predictive performance and a significant reduction in Code Blue events. Specifically, VC-MAES exhibited a reduced pLOS and a lower number of AEs compared with the silent period, along with an increased frequency of early interventions.

### Bridging the Afferent Limb With AI-EWS

Globally, the importance of RRS as a means of preventing AEs has been increasingly emphasized. However, rescue failures may occur even in environments where RRSs are implemented owing to challenges in the afferent limb [19]. The afferent limb refers to the “recognition” phase, encompassing processes such as monitoring, identification, and triggering a response [20]. VC supports automated monitoring, aids in the detection of early high-risk patients, and offers potential solutions for delaying and preventing Code Blue events [1]. In this regard, it can be used systematically and efficiently in environments where an RRS is already established. Conversely, it can maximize the potential impact by addressing unmet needs, even in hospitals where the RRS is not organized or is understaffed.

### Optimizing Care With Predictive Monitoring

In practical settings, the initial assessment and recognition of a patient’s condition are typically performed by nursing staff. Subsequently, the attending physician is notified of the patient’s condition to facilitate decision-making regarding diagnosis and treatment. Vital signs and laboratory tests are also intermittently monitored, making the automatic interpretation and screening of time-series data a critical clinical requirement. As AEs often occur abruptly, such sequential processes can serve as barriers to timely intervention. VC can address this challenge by providing predictive alerts before the occurrence of AEs. This study confirmed a short-term decrease in UIT and death cases, which was found to have led to a significant long-term reduction in Code Blue events.

VC provides the opportunity to assess a patient’s condition (eg, laboratory tests, blood culture, and imaging) proactively or report it to the attending physician early, enabling the preparation of the necessary resources in advance. Accordingly, our findings suggest that VC implementation leads to notable clinical improvements, including increased early intervention and reduced AEs. Prior studies have indicated that several patients who ultimately experience CA remain in the GW for extended periods before being transferred to the ICU (or are never transferred at all), which negatively affects their survival rates. Consequently, these studies asserted that AI-based EWSs have the potential to reduce in-hospital mortality [15,21-23].

We conducted a subgroup analysis of the frequency of early interventions and observed a more significant increase in the GW following VC use (Table 5). It is well established that early intervention improves patient outcomes [24-26]. The occurrence of any intervention or evaluation within 2 hours of the initial risk prediction of VC can be interpreted as an indication that the clinician agreed with this information and suspected patient deterioration. VC can detect AEs more quickly than traditional scoring systems, thereby offering the benefit of securing opportunities for early recognition and treatment, and potentially preventing AEs in the GW, where closed observation may not be as rigorous. Conversely, as patients in the ICU were already under real-time close observation by medical staff, there was no change in the intervention rate within 2 hours of the first elevation. Similarly, a trend was observed in which the difference in intervention rates diminished as the patient severity increased. These results were predictable and reasonable. Based on these findings, we propose that the use of VC-MORS in the ICU is more beneficial for real-time patient monitoring, enabling the early prediction of critical events with high predictive power, rather than offering new information that could lead to additional prescriptions.

In conclusion, VC serves as a clinical decision-supporting system that provides supplementary information for health care providers rather than functioning as an independent diagnostic tool. Ultimately, the clinical judgment of health care professionals remains the final determinant in decision-making. The successful integration and adaptation of AI-EWS into clinical practice suggest the potential for clinical decision-supporting systems to be used more extensively. Various research will be needed to achieve this. We believe that the synergy between the enhancement of health care professionals’ ability to use predictive information and the advancement of AI-EWS could yield significant outcomes for patients and clinical settings.

This study demonstrates the effectiveness of AI-based EWS in a community hospital without an RRS; however, its applicability to institutions in resource-limited settings requires further consideration. The adoption and integration of health AI can vary significantly across countries, depending on geopolitical and socioeconomic development [27,28]. Consequently, several challenges may arise in rural hospitals or institutions with limited digital infrastructure [29]. These include the absence of real-time EMR systems, poor clinical data quality, limited clinician training in AI tools, and variability in clinical workflows and institutional acceptance. While simplified models may offer a feasible alternative, future research should explore implementation strategies for lightweight AI-EWS systems and scalable integration approaches in community-based and resource-limited settings.

### Limitations

This study has several limitations. First, the control period was relatively short owing to constraints in data extraction following the computerized system upgrade period at our institution. A comparative analysis was conducted within a given period using the same computerized system. Second, the effect of VC on patient prognosis following discharge was not investigated. Third, although this was a real-time EWS validation study, it did not evaluate interaction variables between AI-based EWS and the clinician, such as alarm fatigue. Given that real-time EWS operates as a continuous monitoring system, some degree of alert fatigue may perhaps be unavoidable. While adjusting thresholds—such as lowering sensitivity—may mitigate excessive alerts, it may also increase the risk of missing critical events. Future research will focus on assessing the effectiveness of clinician-AI interactions through the AI-based EWS and their influence on discharge outcomes and postdischarge prognosis.

### Conclusions

A well-designed and validated AI-based EWS, such as VC, can effectively reduce major in-hospital AEs by enabling early detection and intervention. By supporting clinical decision-making without replacing professional judgment, this study demonstrates the potential of AI-EWS to enhance patient outcomes and optimize resource utilization in hospital settings.

## Abbreviations

AE: adverse event
AI: artificial intelligence
APACHE II: Acute Physiology and Chronic Health Evaluation II
AUPRC: area under the precision-recall curve
AUROC: area under the receiver operating characteristic curve
CA: cardiopulmonary arrest
EMR: electronic medical record
EWS: early warning system
GW: general ward
ICU: intensive care unit
MAES: Major Adverse Event Score
MEWS: Modified Early Warning Score
NEWS: National Early Warning Score
pLOS: prolonged length of stay
RRS: rapid response system
SOFA: Sequential Organ Failure Assessment
UIT: unplanned ward-to-intensive care unit transfer
VC: VitalCare
